# The effect of running experience and speed on local dynamic stability in running

**DOI:** 10.3389/fspor.2025.1387934

**Published:** 2025-04-03

**Authors:** Adrien Cerrito, Larissa Wittwer, Kai-Uwe Schmitt

**Affiliations:** ^1^ Academic-Practice-Partnership Between School of Health Professions at Bern University of Applied Sciences and University Hospital of Bern, Bern, Switzerland; ^2^ Movecenter Physiotherapy, Feldmeilen, Switzerland

**Keywords:** running, local dynamic stability, kinematics, lower extremities, motor control

## Abstract

**Introduction:**

As a coach or health care professional working with physically active people, it can be difficult to guide runners towards safe and effective progression, because making changes to single aspects of running technique may not lead to the desired result. Alternatively, it has been proposed to consider the human body as one complex system when assessing and improving human movement. From this perspective, it appears that the movement variability and local dynamic stability, expressed as the maximum Lyapunov exponent (LyE_max_), may be of particular interest. This study investigated the difference in LyE_max_ of the lower extremities' main joints (ankle, knee, hip) between experienced and novice runners at different running speeds.

**Methods:**

Thirty-six participants were recruited, with 18 experienced runners and 18 novice runners. Participants ran at three different speeds on a treadmill for 10 minutes in each of the following conditions: preferred running speed, 10% slower, and 10% faster. Twenty-six participants were included in the analysis. The LyE_max_ was calculated from joint kinematics and a two-way ANOVA with repeated measures was applied.

**Results:**

The results showed that there is a strong effect of running experience on the LyE_max_ with the experienced runners having more local dynamic stability. The effect of running speed was statistically significant only for the hip joint, where higher running speeds resulted in more local dynamic stability in both groups.

**Discussion and conclusion:**

The results should be interpreted with caution, particularly due to the low running speeds chosen by the novice runners. Nevertheless, the study's findings support the emerging view that movement variability is a parameter on which it is important to focus, and that local dynamic stability should be improved in novice runners as well as with athletes or patients who are in a return-to-sport phase of a rehabilitation.

## 1 Introduction

Running is a natural way of locomotion that represents a form of physical activity practiced by millions of individuals worldwide [e.g., (1–3)]. Running is appreciated for multiple reasons, such as its simplicity, accessibility, and convenience. In addition, it has many health benefits, for instance providing a protective effect against chronic diseases and premature death from any condition (4), and improving mental health (5). But while some health benefits might have immediate effect, e.g., for mental health (6), to exploit most other benefits, running must be practiced regularly and for a prolonged period. During this period, not only physiological adaptations take place (7), but experience is also gained and running technique changes. For example, experienced runners generally appear to adopt techniques with shorter steps and higher step frequency, compared to inexperienced (i.e., novice) runners (8). Also, differences in joint angle magnitudes, such as greater amplitudes in ankle inversion/eversion and hip abduction/adduction in novice runners, were found in previous studies (9). Kinetic differences were also reported, with greater ankle inversion and hip abduction moments in novice runners (9). These differences may be linked to the development of running-related injuries or to lower running economy and could partially explain higher risk of injury (10) and lower performance in novice runners. Nevertheless, applying these data in coaching, prevention or rehabilitation activities as a guide to rapidly and safely progress in running remains difficult, because it would imply the existence of one perfect running technique. In fact, sports techniques are likely to be highly individual (11) and in running, differences in anthropometry, preferred muscle activation patterns and functional statuses (e.g., joint mobility) could be sufficient to explain uniqueness in gait patterns. Hence, making changes to the running technique to improve performance and prevent running-related injuries should be an individualised operation rather than rely on averaged biomechanical data from single body segments, joints or spatio-temporal parameters (12).

In contrast, perceiving the body as one complex entity might provide deeper insight into the issue, because the output of the interactions between all body parts is considered rather than the body parts individually. Approaches stemming from the complex systems theory, such as nonlinear movement analysis, have been proposed to this end (13). More specifically, movement variability is thought to reflect how the central nervous system adapts motor strategies to internal and external perturbations and to carry important information about the health and expertise status of the motor behaviour (14). In this context, it appears that there is an optimum lying between too little and too much movement variability. To safely progress in running, it is conceivable that it is more important to achieve or maintain this state, than to improve certain movements in isolated joints according to reference values. In particular, a system aiming for balanced movement variability requires local dynamic stability—often expressed as the maximum Lyapunov Exponent (LyE_max_)—to avoid its collapse. It has been shown that the level of local dynamic stability differed between groups of various experience levels, with experienced runners generally showing more local dynamic stability than novice runners (15, 16) and factors such as footwear (17) and exhaustion (16) having substantial influence. These factors may represent important training variables that can be manipulated when working with runners in practice. Nevertheless, one aspect that has not been investigated is the difference in local dynamic stability between experienced and novice runners when changing running speed. This is a significant aspect because varying running speeds represents an important part of running training to prevent monotony (18, 19), thereby ensuring progress and expand the richness of motor repertoire.

Hence, this study's aim was to investigate the effect of the level of experience and running speed on local dynamic stability expressed as LyE_max_ in the ankle, knee, and hip joints in the sagittal plane. The ankle joint served as the primary reference for the analysis. The focus was set on local dynamic stability of joint kinematics rather than kinetics for feasibility reasons. The latter requires more technical equipment, which may strongly limit the transferability into coaching or clinical practice. Moreover, the sagittal plane was chosen for the analysis due to its high level of reliability that is also found in portable measurement systems, such as inertial sensors (20), which can easily be used by coaches or clinicians.

## 2 Materials and methods

A cross-sectional study was conducted that compared a group of experienced runners (Exp) with a group of novice runners (Nov) running at three different speeds: preferred running speed (“Preferred”), 10% faster (“Fast”) and 10% slower (“Slow”).

### 2.1 Participants and ethics

Precise sample size calculation was difficult to perform because, to the best of the authors' knowledge, no prior study investigating differences in local dynamic stability between novice and experienced runners in different running speed conditions existed. Nevertheless, some work investigating the inverse of stability, i.e., movement variability expressed in terms of coordination variability, was found (21), on which basis a medium effect size was expected. Sample size estimation was therefore performed using a Cohen's f ranging from 0.2 to 0.25, as well as an α and β error probabilities of 0.05 and 0.2, respectively. The calculations performed in G*Power (v. 3.1) (22) resulted in a sample size of 28–44 participants and a sample size of 36 was chosen for this study as the middle value.

Thirty-six participants were recruited using the following eligibility criteria. Basic criteria applied to both groups: (i) feeling healthy, (ii) being free of any pain symptoms, and (iii) being aged between 20 and 40 years of age. Group-specific inclusion criteria were also set. For the Exp group, participants with a minimum of 4 years of running experience with a minimum weekly training volume of 20 km on a monthly average were eligible. For the Nov group, participants with a running experience ranging from none to a maximum of 6 months were eligible. Exclusion criteria were the following: (i) musculoskeletal or neurological disorders that could affect movement patterns, (ii) musculoskeletal injury in the past 12 months, (iii) serious medical conditions (e.g., tumor, myocardial infarction), and (iv) running experience matching neither group requirements.

Ten participants had to be excluded from the analysis due to technical problems in the kinematic data, such as marker detachment or occlusion, leading to a final sample size of 26. Consequently, a *post hoc* power analysis (1-β) of the results was performed. Demographic data, anthropometry, and training characteristics are presented for both groups together as well as separately in Table 1.

**Table 1:** Sample characteristics.

	Overall	Exp	Nov
Sample size	*N* = 26	*n* = 13	*n* = 13
Male/Female [*n*]	14/12	7/6	7/6
Age [years]	30.9 ± 3.5	30.6 ± 3.7	31.2 ± 3.5
Height [cm]	176.1 ± 9.1	175.7 ± 10.2	176.5 ± 8.3
Weight [kg]	68.9 ± 11.8	65.7 ± 9.6	72.0 ± 13.4
Preferred running speed [km/h]	8.0 ± 1.8	10.2 ± 1.7	7.5 ± 1.1

The study was conducted in line with the Declaration of Helsinki and it was approved by the responsible Ethics Committee of the Canton of Bern (Ref.: 2022-01136). All participants provided written informed consent to participate.

### 2.2 Data acquisition and apparatus

Basic demographic, health- and training-related data were collected through a custom questionnaire delivered via LimeSurvey (v. 2.56.1, LimeSurvey, Hamburg, Germany). Anthropometrics (body height and weight) were measured using a stadiometer and a scale. Running kinematics were obtained from a 16-camera optoelectronic motion capture system and the Vicon Nexus Software (Vicon, Oxford, UK). For that purpose, 28 retroreflective markers were attached to participants' anatomical landmarks according to the Conventional Gait Model 2.3 for the lower limbs (23). Kinematic data were collected at 200 Hz. Participants ran on a treadmill (h/p/cosmos Quasar Med, h/p/cosmos sports medical gmbh, Nussdorf-Traunstein, Germany) with 0% incline in three trials at different speeds.

### 2.3 Experimental procedure

**Figure 1:**
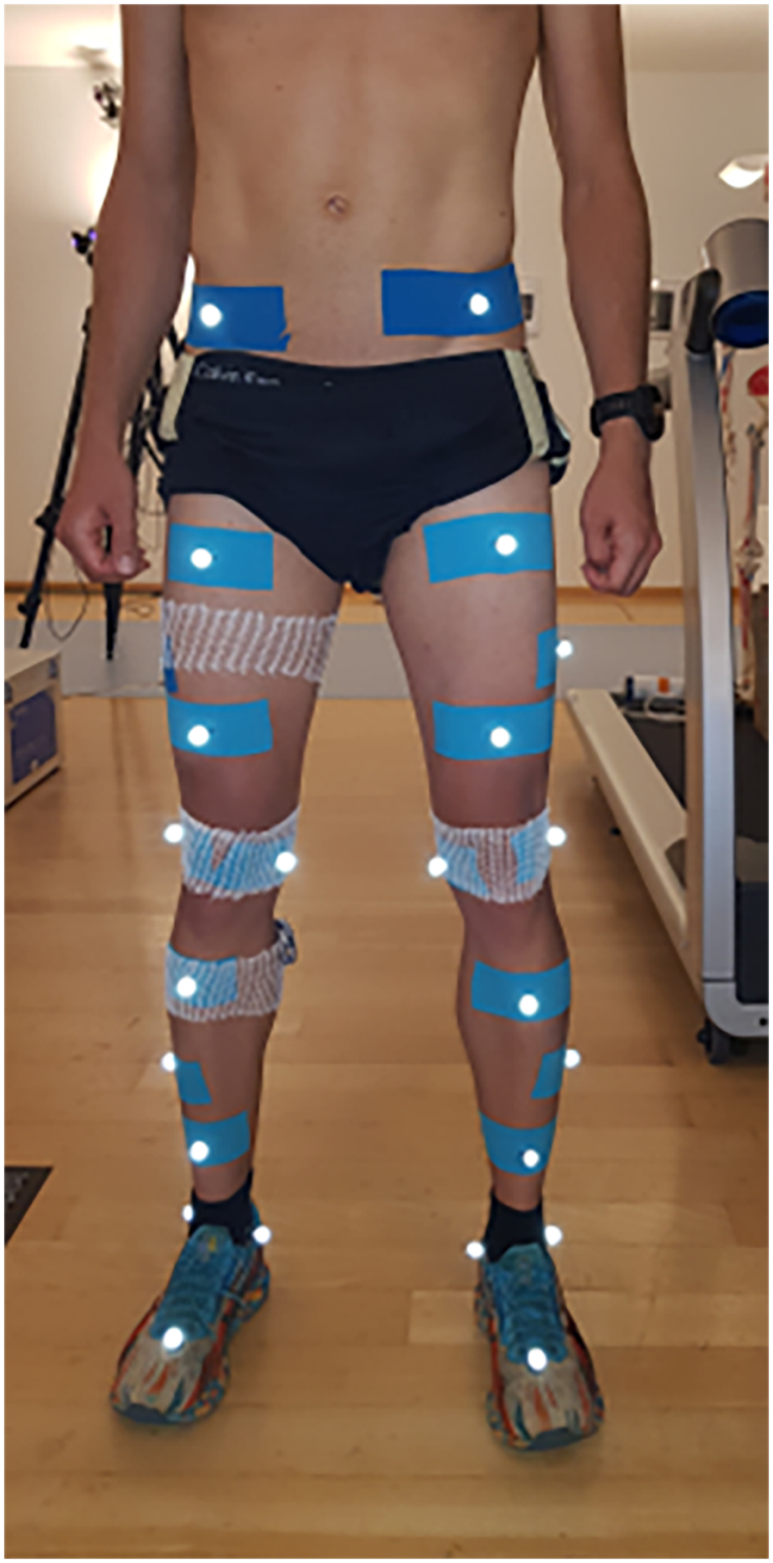
Marker set up used: conventional gait model 2.3 for the lower limbs.

Participants were invited for a single data collection session. Upon arrival, written informed consent was obtained and participants were asked to wear sports clothes and their own running shoes. Data collection began with the administration of the questionnaire and with taking the anthropometric measures. Then, participants were equipped with the markers, which were secured with double-sided tape and “kinesiology tape” (Figure 1). They were asked to draw opaque envelopes to randomly set the order of the three running speeds Slow, Preferred, and Fast. Participants then warmed up according to a standardised routine consisting of squats-to-calf raises, squat-lunges, and two running drills. Then, they were asked to further warm up on the treadmill, to familiarise with it, and to select their preferred running speed. For this purpose, they were asked to try various speeds and to find the one they would choose for a comfortable long run (24). Preferred running speed was written down, the 10% were calculated and subtracted (Slow) and added (Fast) to it, and running trials were started according to the previously randomised order. In each running trial, participants ran for 10 min, and the last 2 min were recorded. Between each trial, participants had a break of 3–5 min.

### 2.4 Data processing

#### 2.4.1 Kinematic data

Kinematic data were cleaned within Vicon Nexus, which included marker labelling, gap filling and removing ghost markers. Joint angles from the CGM2.3 model were also calculated via Vicon Nexus. All further data processing was performed within MATLAB (v. R2021b, Mathworks, Natick, MA, USA) using custom-written scripts. Joint angle data were low-pass filtered using a 2nd order zero-lag Butterworth filter with a cut-off frequency of 15 Hz. The cut-off frequency was set based on a visual inspection of the Power Spectrum Analysis performed with pilot data from two other studies (25, 26). The trials were then cropped to a standardized length containing the first 100 complete strides (27). The number of strides used was rounded up from the recommendations by Raffalt et al. (27) and Mehdizadeh and Sanjari (28). Stride detection was based on kinematic data, where peak knee flexions were detected to separate strides. Visual quality inspection was performed to ensure correctness of this process.

#### 2.4.2 Maximum Lyapunov exponent

In brief, the LyE_max_ quantifies the level of chaos in a dynamical system (i.e., a system that evolves over time). Analysing running gait using an approach based on dynamical systems theory allows the application of the LyE_max_ method to quantify how well the motor system can attenuate perturbations to maintain stable running gait pattern (13). For the analysis, the time series data (i.e., kinematics) are reconstructed into a state space to reveal information about the underlying dynamic process. An optimal time-lag (*τ*) and embedding dimension (*d*) must first be identified to generate an appropriate state space and allow optimal unfolding of new information about the underlying dynamical process. In this higher-dimensional state space, the time series data are expected to form trajectories orbiting an attractor. The LyE_max_ effectively quantifies the rate at which these orbiting trajectories diverge, where higher values indicate less capability of the motor system to attenuate perturbations (13, 29). The interested reader is referred to Stergiou (13) for more comprehensive description.

All calculations for the LyE_max_ were performed in MATLAB using adapted scripts from the NONANToolbox (30). First, to perform the state-space reconstruction, the time-lag *τ* and the embedding dimension *d* were computed using the Average Mutual Information (AMI) and the False Nearest Neighbour (FNN) methods, respectively (13). For AMI, a maximum time-lag of one second was set. The calculation of AMI and FNN was performed for all participants in all conditions for the ankle joint kinematics in the sagittal plane. These values were averaged to obtain mean *τ* and mean *m*, which were then used to calculate LyE for the ankle, knee and hip joint kinematics in the sagittal plane (27). The LyE_max_ was calculated with the Wolf algorithm (27) using an evolve parameter of 0.05 times the sampling frequency (13).

### 2.5 Statistical analysis

Statistical analysis was performed in Jamovi (v. 2.3.28) (31). First, the questionnaire data and the three running speeds were analysed using descriptive statistics [mean ± standard deviation (SD)]. The analysis of the effect of experience level and running speed on LyE_max_ was then calculated using a two-way ANOVA with repeated measures. Normal distribution of residuals was verified visually using a histogram and Q-Q plot. In addition, a Shapiro–Wilk test was performed to further check for normal distribution. Sphericity was tested using Mauchly Test. All tests were performed with a significance level of 0.05. Where the ANOVA indicated statistically significant differences, pairwise comparisons were performed with Bonferroni adjustment. Effect sizes were calculated as partial eta squared (*η*_p_^2^) and interpreted as follows: 0.01: small effect; 0.06: medium effect; 0.14: large effect (32).

## 3 Results

### 3.1 Participants' training and injury characteristics

**Figure 2:**
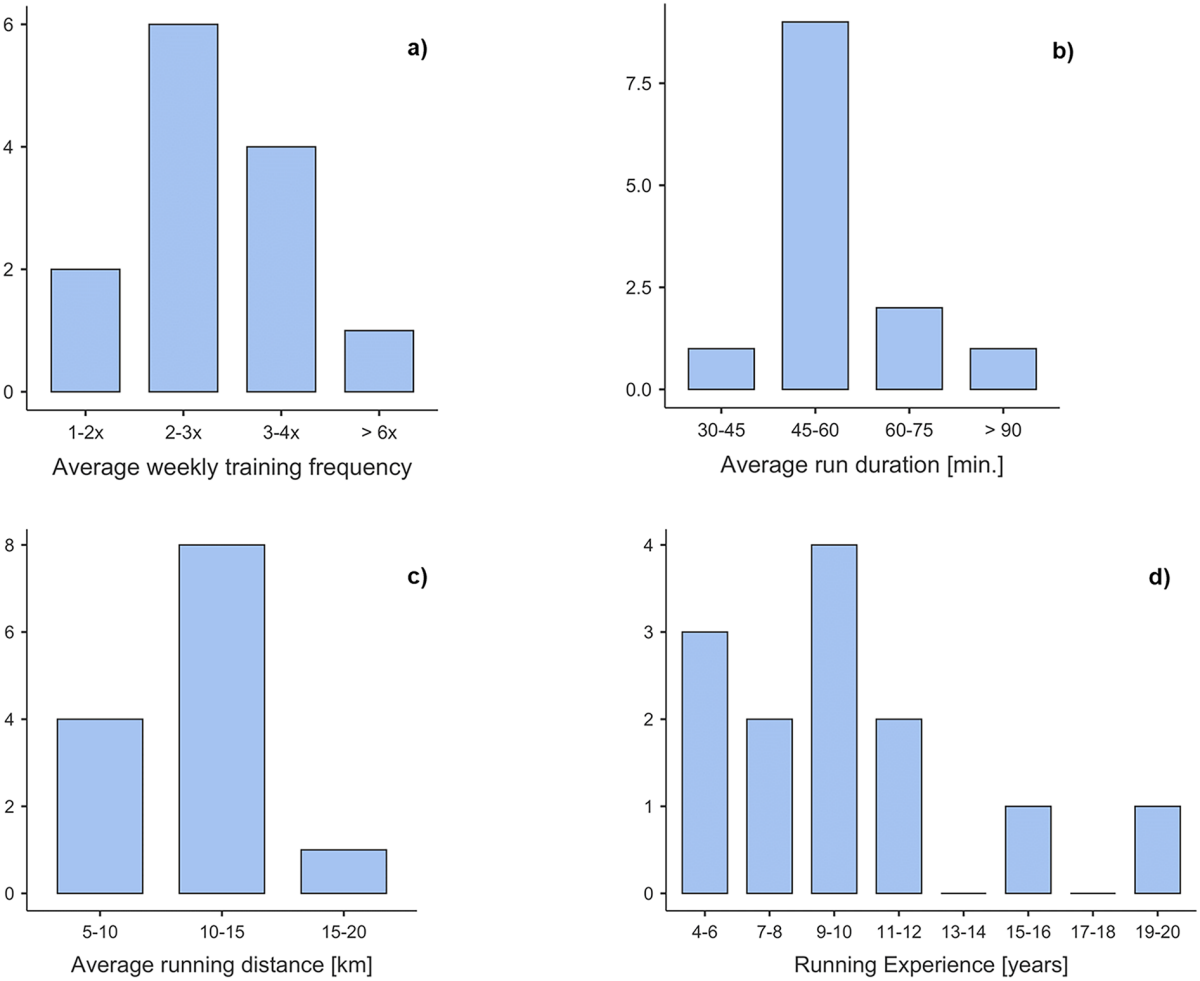
Training-related data of the Exp group. The top left bar plot **(a)** presents the distribution of the average weekly training frequency, **(b)** the average duration of run training sessions, **(c)** the average running distance per running training, and **(d)** the running experience.

Training characteristics of the Exp group are presented in Figure 2. Six out of 13 Exp runners (46.2%) indicated being member of a running group and 12 participating in competitions (92.3%). All Exp runners participated in other sports, such as swimming, cycling, pilates, or volleyball. Six of the Nov runners (46.2%) were physically active on a regular basis by participating in one or multiple sports (e.g., swimming, yoga, hiking, or badminton).

Out of the Nov and Exp groups, three runners (23.1%) and one runner (7.7%), respectively, reported having sustained an injury to the lower extremities from which they did not recover fully. In the Nov group, injuries occurred between nine months and six years prior to data collection and the recovery ranged from 80% to 95%. In the Exp group, the injury occurred three years prior to data collection and the runner reported a 95% recovery.

### 3.2 LyE_max_ calculations

The AMI and FNN calculations resulted in a time-lag *τ* of 29 frames and an embedding dimension *d* of 6.

### 3.3 The effect of running experience, running speed and their interaction of the LyE_max_

**Figure 3:**
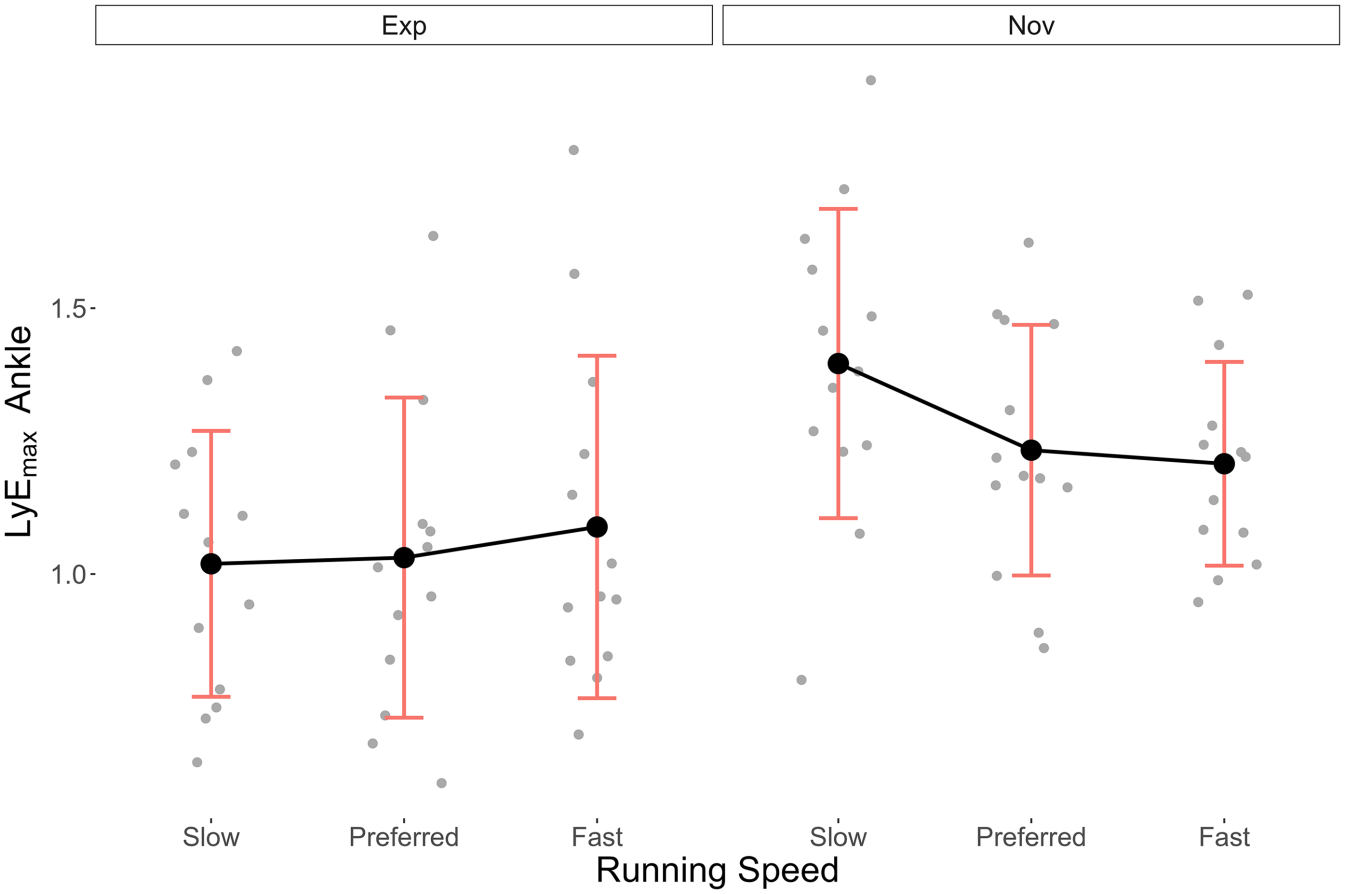
Mean ± SD LyE_max_ data of the ankle joint in the Exp and Nov groups at different running speeds.

**Figure 4:**
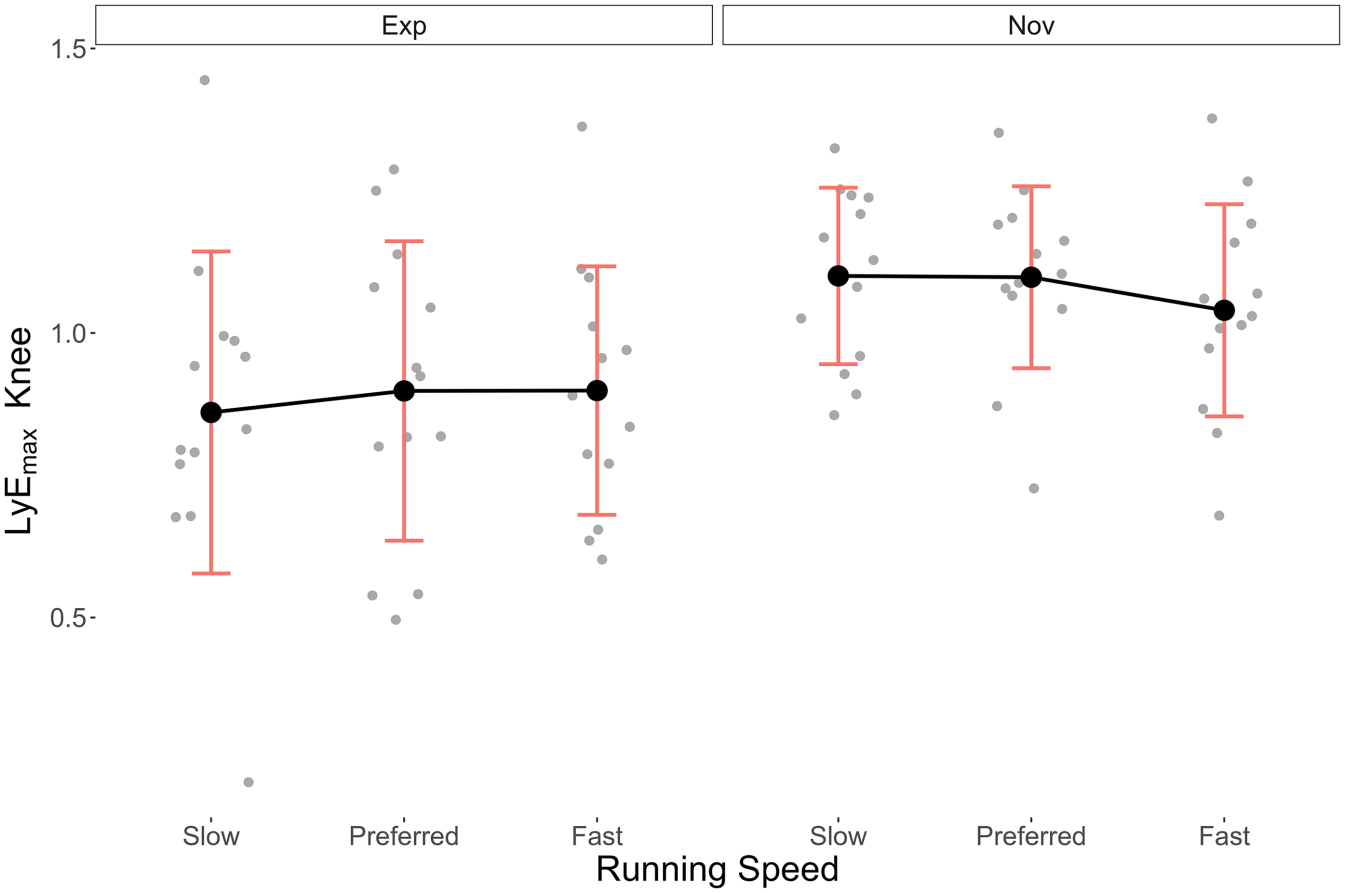
Mean ± SD LyE_max_ data of the knee joint in the Exp and Nov groups at different running speeds.

**Figure 5:**
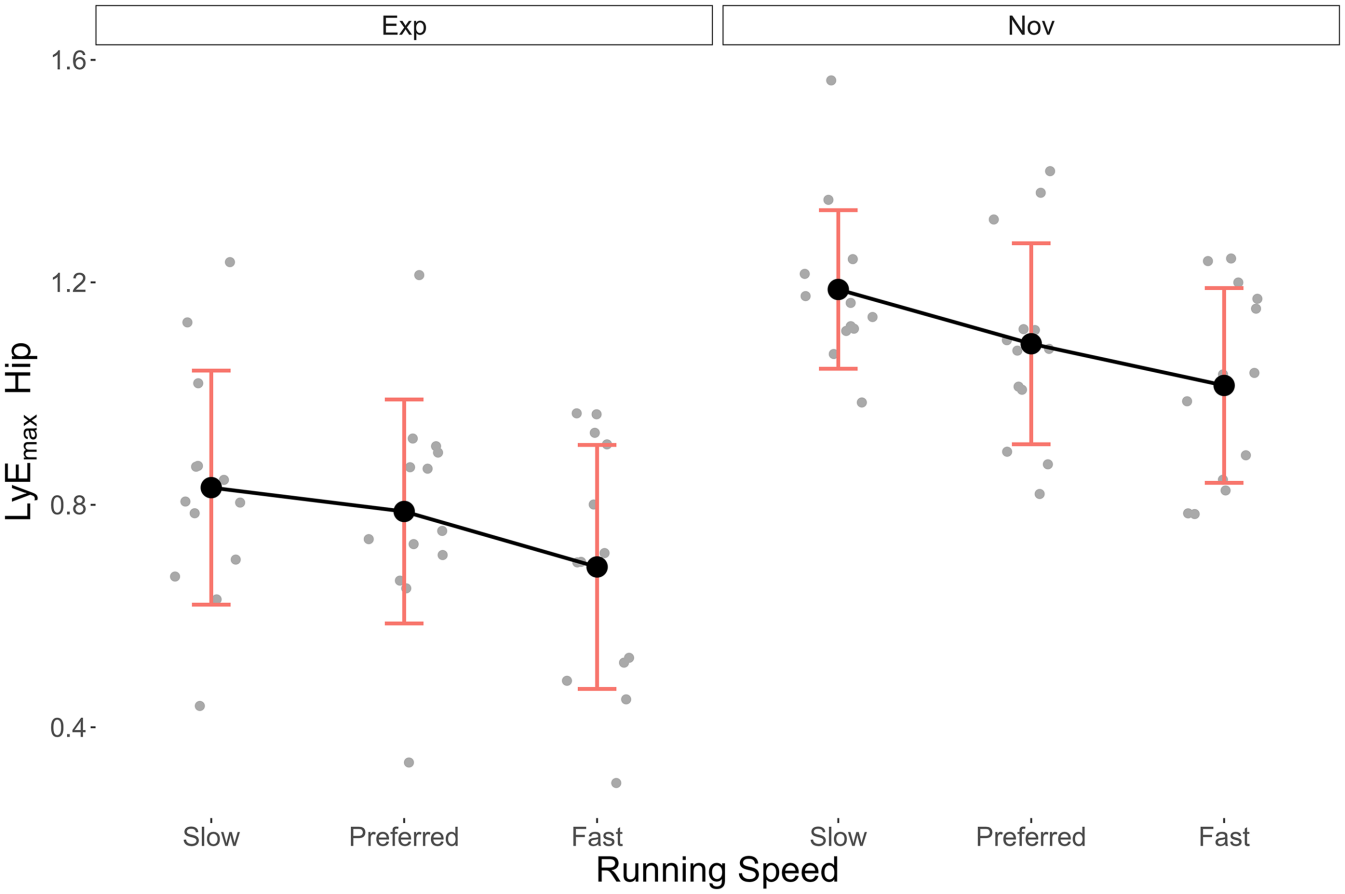
Mean ± SD LyE_max_ data of the hip joint in the Exp and Nov groups at different running speeds.

Table 2 presents the mean LyE_max_ (±SD) for the ankle, knee and hip joints at each running speed for both groups. Figures 3–5 illustrate the distribution of these data, respectively.

**Table 2:** Mean ± SD LyE_max_ values for the ankle, knee, and hip joints at fast, preferred, and slow running speeds for experienced (Exp) and novice (Nov) runners.

	Fast	Preferred	Slow
Ankle			
Exp	1.09 ± 0.32	1.03 ± 0.30	1.03 ± 0.25
Nov	1.21 ± 0.19	1.23 ± 0.24	1.40 ± 0.29
Knee			
Exp	0.90 ± 0.22	0.90 ± 026	0.86 ± 0.28
Nov	1.04 ± 0.19	1.10 ± 0.16	1.10 ± 0.16
Hip			
Exp	0.69 ± 0.22	0.79 ± 0.20	0.83 ± 0.21
Nov	1.02 ± 0.18	1.09 ± 0.18	1.19 ± 0.14

For the ankle joint, no assumption for the ANOVA calculation was violated (Shapiro–Wilk test *p* = 0.38, Mauchly test *p* = 0.61). Mean LyE_max_ (±SD) was consistently lower in the Exp than in the Nov group, with values for Fast, Preferred, and Slow of 1.09 (±0.32), 1.03 (±0.30), and 1.02 (±0.25) and 1.21 (±0.19), 1.23 (±0.24), and 1.40 (±0.29), respectively. The ANOVA revealed a statistically significant effect of running experience on LyE_max_ (*p* < 0.02, 1-*β* = 65%) and that this effect is large (*η*_p_^2^ = 0.22). In contrast, while the effect of running speed alone appeared to be medium (*η*_p_^2^ = 0.06), it was not statistically significant (*p* = 0.26, 1-*β* = 18%). The interaction between running experience and running speed, however, showed large effect on the LyE_max_ (*η*_p_^2^ = 0.14) and was statistically significant (*p* < 0.03, 1-*β* = 41%). The results of the *post hoc* pairwise comparison Bonferroni tests showed non-significant *p*-values in all comparisons (*p* = 0.48–1.00) for the effect of running speed on LyE_max_ in the ankle. For the interaction effect, the results showed that the LyE_max_ differed between Exp and Nov particularly in the Slow running speed condition (*p* = 0.03) and where Nov run Slow and Exp run Fast (*p* = 0.03).

For the knee and hip joints, normal distribution of the model residuals (Shapiro–Wilk test *p* = 0.09 and *p* = 0.43, respectively) and sphericity (Mauchly test *p* = 0.29 and *p* = 0.12, respectively) was also assumed. Here too, mean LyE_max_ (±SD) was consistently lower in the Exp group. For the knee in the Fast, Preferred, and Slow conditions, values were 0.90 (±0.22), 0.90 (±0.26), and 0.86 (±0.28), respectively, in the Exp group, and 1.04 (±0.19), 1.10 (±0.16), and 1.10 (±0.16) in the Nov group. The ANOVA showed a large, statistically significant effect of running experience on the knee's LyE_max_ (*η*_p_^2^ = 0.24, *p* = 0.01, 1-*β* = 70%). Both the effect of running speed and the interaction between the two independent variables were small and not statistically significant (*η*_p_^2^ = 0.01, *p* = 0.79, 1-*β* = 7% and *η*_p_^2^ = 0.03, *p* = 0.51, 1-*β* = 11%, respectively). The *post hoc* analysis showed no significant *p*-values, neither for the effect of running speed (*p* = 1.00) nor for the interaction effect (*p* = 0.18–1.00).

For the hip in the Fast, Preferred, and Slow conditions, values were 0.69 (±0.22), 0.79 (±0.20), and 0.83 (±0.21), respectively, in the Exp group, and 1.02 (±0.18), 1.09 (±0.18), and 1.19 (±0.14) in the Nov group. In the ANOVA, both independent variables showed statistically significant large effects on LyE_max_, with running experience *η*_p_^2^ = 0.57 (*p* < 0.01, 1-*β* = 99%) and running speed *η*_p_^2^ = 0.25 (*p* < 0.01, 1-*β* = 73%). The effect of the interaction was very small and not statistically significant (*η*_p_^2^ = 0.01, *p* = 0.79, 1-*β* = 7%). In the *post hoc* analysis, statistically significant results were found in the comparison between Slow and Fast running speed (*p* < 0.01). Regarding the interaction effects, seven pairwise comparisons were statistically significant, with six concerning the effect of running experience at the same (e.g., Slow vs. Slow, *p* < 0.01) or different running speed conditions (e.g., Nov running Slow vs. Exp running Preferred, *p* < 0.01). One comparison within the Nov group showed statistically significant difference in LyE_max_ in Slow vs. Fast (*p* = 0.02).

The *post hoc* pairwise comparisons from the Bonferroni tests for the ankle, knee and hip joints are presented in the Supplementary Material.

## 4 Discussion

This study investigated the effect of running experience and running speed on the local dynamic stability of the ankle, the knee, and the hip joints. The results showed that runners from the Exp group had statistically significantly lower LyE_max_ values in all three joints of the lower limbs in all three running speed conditions compared to those from the Nov group, meaning that running experience has a strong effect (*η*_p_^2^ = 0.22–0.57) on increased local dynamic stability. The results corroborate prior studies that also showed experienced runners exhibit more local dynamic stability in the lower limb's joints while running, although these studies investigated other parameters, such as the effect of footwear (15) or fatigue in a 5 km exhaustive run (16).

In contrast, the running speed condition did not show statistically significant results, except for the hip joint. An analysis of the trends of the LyE_max_ over the three running speed conditions, nevertheless, appeared interesting. For the ankle joint in the Exp group, the LyE_max_ increased from Slow to Fast, with the largest increase occurring between Preferred and Fast. This result is in line with the trends observed by Wang et al. and Aghaie et al. in the knee and hip joints (21, 33). Wang et al. (21) stated that the increase in movement variability with increasing speed could be interpreted in two ways. First, it could signify that faster running speeds demand more variability in movement patterns to increase adaptability to internal and external perturbations. Second, it could reflect less stability as the running speed becomes physically more challenging and leads to difficulties in dynamically stabilising joint movements. Nevertheless, although the authors of the current study agree with Wang et al. (21) that conclusions about the interpretation is difficult at this stage, they believe the first view better explains the results, because in the current study, the running speed conditions were based on the preferred running speed. This means that participants felt relatively comfortable while running even in the Fast condition and that the task probably did not lead to as much biomechanical demands as in sprinting and to as much difficulty in dynamically stabilising the joint movements.

Interestingly, participants of the Nov group exhibited the opposite trend with decreasing LyE_max_ values as the running speed increased. This trend was observed in all three joints, although to a lesser extent in the knee joint. A possible explanation for this result is that the average preferred running speed selected by Nov participants (7.5 km/h) was close to the speed of 7.2 km/h that is considered to be the preferred speed at which unexperienced runners transition from walking to running (34). From a dynamical systems' perspective, such transition phases are always characterised by increased levels of instability (35), which may explain the largest LyE_max_ values found in the Slow and Fast conditions, respectively.

In both groups' hip joints, the results revealed the lowest LyE_max_ values overall and showed a decrease as the running speed increased. In the knee, the values slightly increased from Slow to Preferred in the Exp group and slightly decreased from Preferred to Fast in the Nov group. These hip and knee results are not in line with the findings of Wang et al. and Aghaie et al. who found an increase in movement variability and LyE_max_, both meaning less stability (21, 33). The discrepancies might be due to several reasons. First, Wang et al. (21) did not investigate local dynamic stability but rather movement variability expressed as variations in coordinative patterns, which only allows a gross conceptual comparison to the current study, where variability is the inverse of stability. Second, the authors did not report the number of strides investigated per participant, but the number is likely to be relatively low compared to the current study that used 100 strides. The issue of small number of strides also applies to the study by Aghaie et al. (33) who used 50 strides. Finally, Aghaie et al. (33) used inertial measurement unit sensors to obtain joint angles and the Rosenstein algorithm to calculate the Lyapunov exponent, which makes direct comparison difficult. Such methodological disparities have recently been reported in a systematic review on the application of the Lyapunov exponent to the analysis of human movement performance (29) and to facilitate interpretation of the results, future studies should be more strictly based on reference studies, such as the one by Raffalt et al. (27).

The in-depth analysis of the interaction effect revealed that, although the lower preferred running speed in Nov participants led to smaller speed ranges from Slow to Fast (1.5 km/h) than in Exp participants (2.0 km/h), their gradient in ankle LyE_max_ were much higher (0.19) than in the Exp group (0.07). This increased gradient in LyE_max_ in Nov compared to Exp participants was also found in the knee (0.06 vs. 0.04) and hip (0.18 vs. 0.14) joints, yet to a lesser extent, resulting in statistically non-significance. But beyond the current statistical results, the qualitative analysis indicated unequivocally that experienced runners' local dynamic stability was less affected by changes in running speed than that of novice runners. Moreover, provided the statistical power of the interaction effects in the knee and hip was very low (11% and 7%, respectively), the risk of *β* error was relatively high. Hence, future studies should investigate the interaction effects between running speed and experience more closely.

Future research should overcome the limitations of this study. First, participants were asked to run on a treadmill, which is known to alter gait patterns when compared to overground walking [e.g., (36)]. The reason for using a treadmill was that it allows the use of optoelectronic motion capture, which is considered to be the gold standard in motion capture (37), and to use a large amount of strides continuously. Second, the two groups had extremely different running experience and no intermediate group was included. Having a group of intermediate experience could have provided further insight in the role running experience has on local dynamic stability. Third, the sample used for the data analysis was smaller than the targeted sample size estimation. Fourth, Slow and Fast were separated from the preferred running speed by only 10%. It is conceivable that more pronounced results could have been achieved with running speed conditions of 20% lower and higher than the preferred running speed. Moreover, it can be criticised that the preferred running speed is subjective and may not be reliable, especially when determined on a treadmill [e.g., (38)]. This method resulted in preferred running speeds close to the walk-to-running transition [7.2 km/h (39)], thereby limiting the range of applicability of the results. Hence, future studies could consider other possible running speed determination methods, such one based on the Froude number (40) or physiological parameters [e.g., ventilatory threshold (41)], to decrease the subjective aspect. Fifth, parameters such as fatigue after each running bout, menstrual cycle status for female runners, or footwear type were not controlled. These are parameters could have affected the findings of this study and should be included in future studies' protocols. Finally, applying a longitudinal study design should be considered in future to better understand the evolution of local dynamic stability and/or its role in the development of running-related injuries. This is particularly important since the development on injuries is multifactorial (42).

In contrast, the study's strengths were, that the design was optimised to reduce the risk of bias in the calculation of the LyE_max_ and explicitly based on the study by Raffalt et al. (27). This included, for example, the choice of a high number of strides. The sample was also well balanced. For example, both groups were of equal size and had the same sex distribution. It should be noted, however, that there were two more participants in the Nov group that had sustained an injury from which they did not recover fully. This might have slightly biased the results.

Nevertheless, the study's results fit well in the bigger picture that begins to arise from the increased use of nonlinear analysis methods to investigate human movement in relation to performance and motor expertise (43, 44). Briefly, experienced runners are thought to have richer repertoires in movement strategies and are therefore more capable of compensating for internal and external perturbations and ultimately to maintain local dynamic stability (14). This motor capacity is also thought to be related to health status, with insufficient stability or exaggerated variability increasing the risk of developing musculoskeletal injuries, which could explain at least partly why novice runners are at considerably higher risk for running-related injuries than experienced runners (10).

The study has therefore clinical implications that can be of interest to coaches and health care practitioners who work with athletes or physically active patients. In particular, when supervising a novice runner or when in a return-to-sport phase of a runner's rehabilitation, it appears to be important to achieve or retrieve optimal levels of local dynamic stability. This can be performed in parallel to the development of other physical capacities, such as strength training, but might be more important in first place compared to being able to run a certain distance at a certain speed. Achieving such skills could specifically be practiced, for example, by using nonlinear pedagogy methods (45). In particular, varying the running speeds in training might be of particular benefit to train local dynamic stability under varying conditions and become less affected by them, such as the runners from the Exp group of this study.

In conclusion, this study showed that experienced runners have better local dynamic stability in the lower limbs' sagittal kinematics than novice runners. Moreover, this difference is present at preferred running speed, but also in slower and faster speeds. These findings support the view that achieving optimal levels of local dynamic stability is important in sports and clinical practice to safely progress in running.

## Data Availability

The datasets presented in this article are not readily available due to ethical reason data is not available. Requests to access the datasets should be directed to adrien.cerrito@bfh.ch.
