# Supplementary material for: The effect of running experience and speed on local dynamic stability in running
Source: Front Sports Act Living. 2025 Apr 3;7:1387934. doi: 10.3389/fspor.2025.1387934 (PMC12003351; doi:10.3389/fspor.2025.1387934)
Supplement: Supplementary file 1 [file Datasheet1.docx]

# Supplementary material

1. **Ankle**


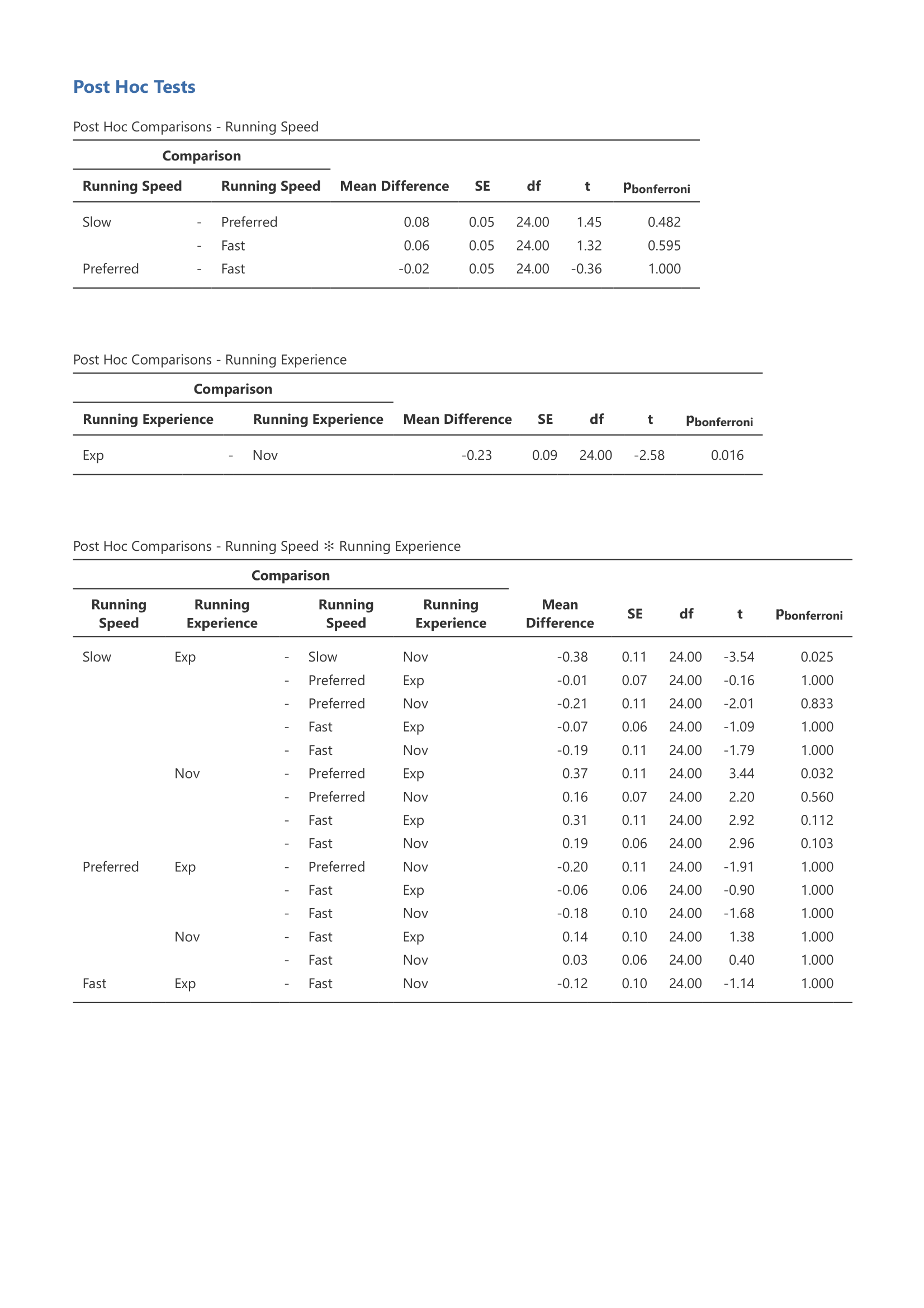


Supplementary material 1: Post hoc analysis of the ankle data. The upper table contains the results of the within-participant effect (running speed condition), the middle table that of the between-participant effect (running experience) and the lower table those of the interaction effects.

1. **Knee**


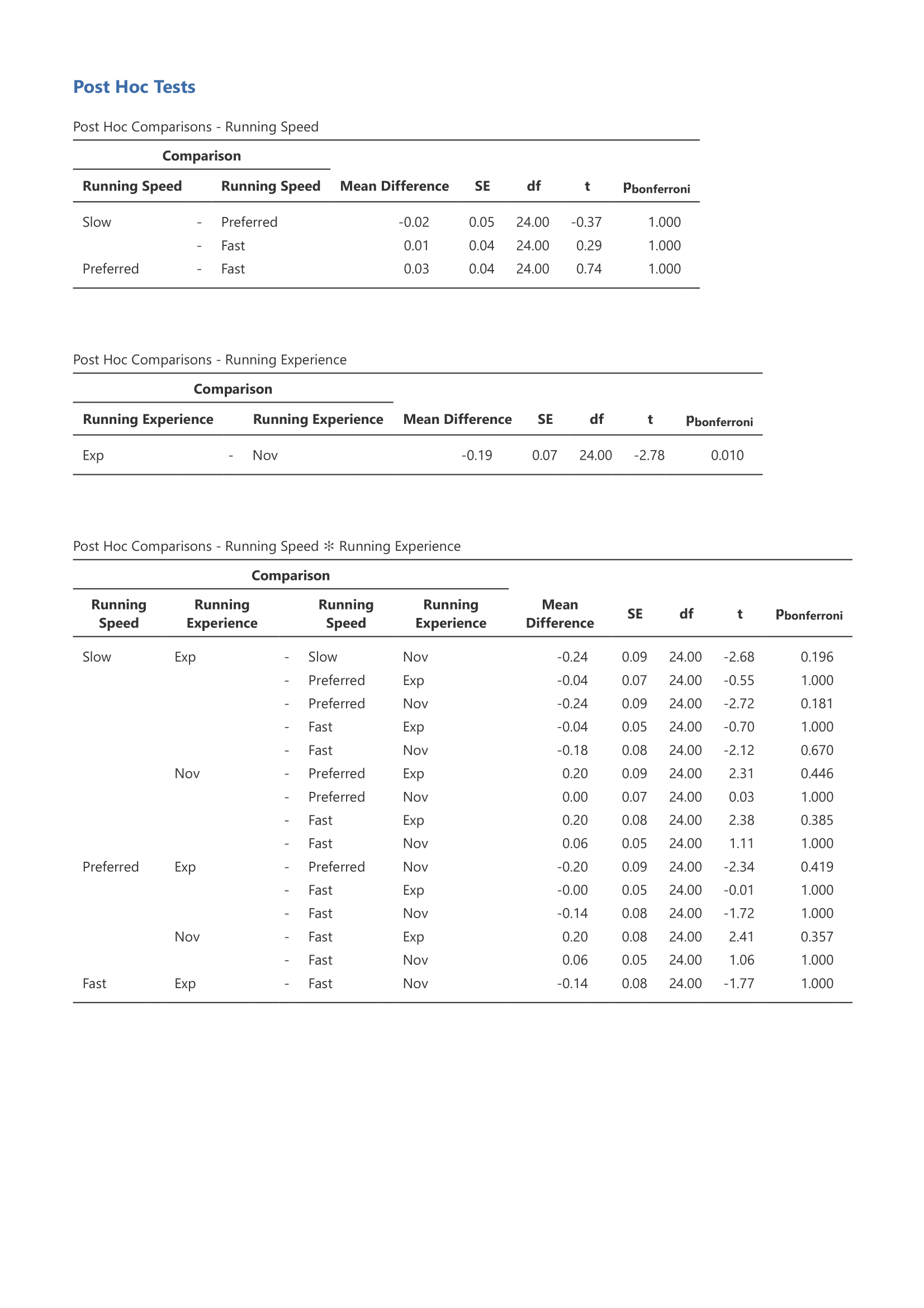


Supplementary material 2: Post hoc analysis of the knee data. The upper table contains the results of the within-participant effect (running speed condition), the middle table that of the between-participant effect (running experience) and the lower table those of the interaction effects.

1. **Hip**


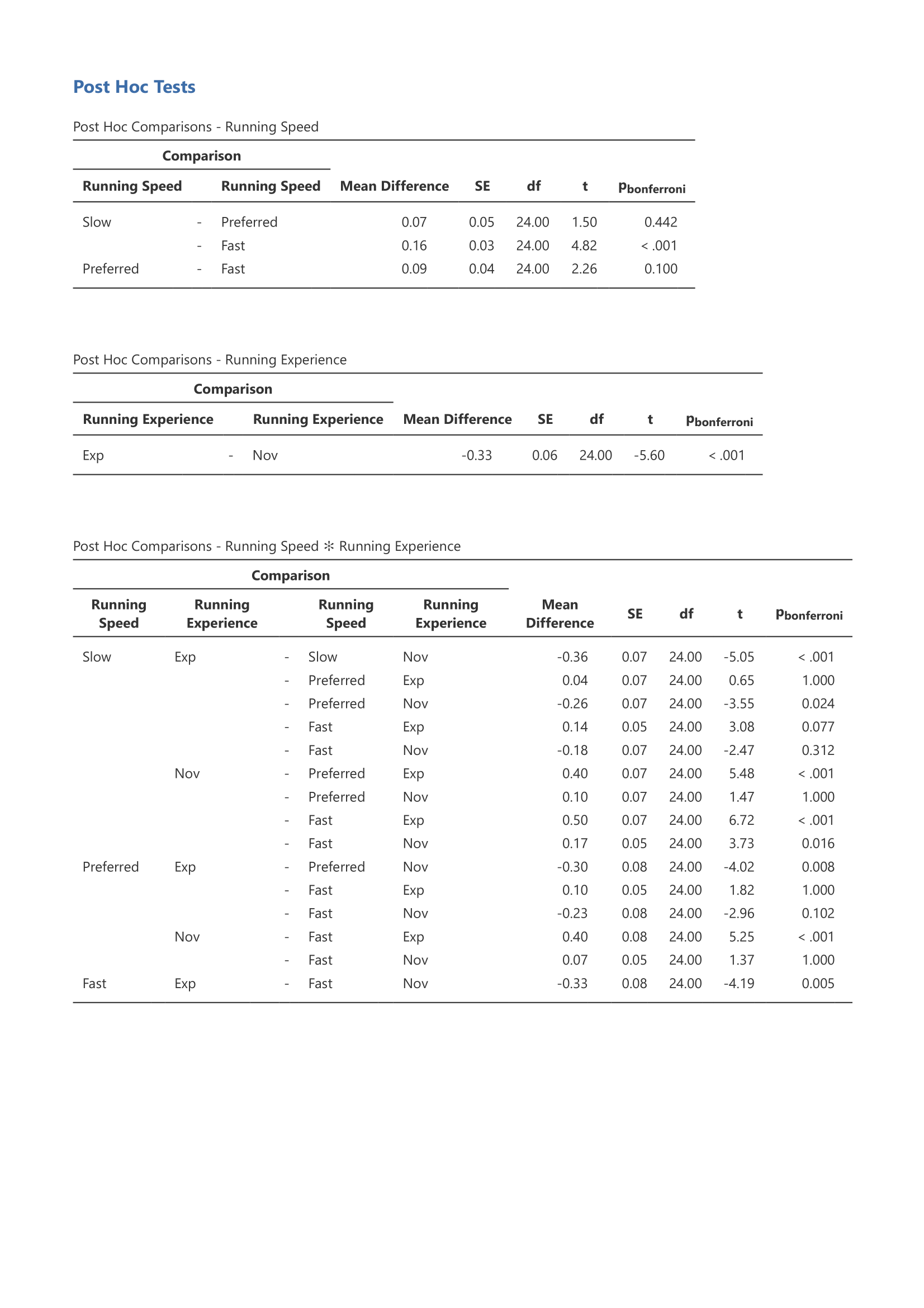


Supplementary material 3: Post hoc analysis of the hip data. The upper table contains the results of the within-participant effect (running speed condition), the middle table that of the between-participant effect (running experience) and the lower table those of the interaction effects.
